# Analysis of Real-World Implementation of the Biopsychosocial Approach to Healthcare: Evidence From a Combination of Qualitative and Quantitative Methods

**DOI:** 10.3389/fpsyt.2021.725596

**Published:** 2021-10-26

**Authors:** Xiaohua Xiao, Haidong Song, Tian Sang, Zhihua Wu, Ying Xie, Qian Yang

**Affiliations:** ^1^School of Public Health, The Children's Hospital, and National Clinical Research Center for Child Health, School of Medicine, Zhejiang University, Hangzhou, China; ^2^Affiliated Mental Health Center Zhejiang University School of Medicine (Hangzhou Seventh People's Hospital), The 4th Clinical College of Zhejiang Chinese Medical University, Hangzhou, China; ^3^Women's Hospital, Zhejiang University School of Medicine, Hangzhou, China; ^4^School of Public Health, Zhejiang University, Hangzhou, China

**Keywords:** biopsychosocial approach, healthcare, medical staff, psychosocial status, qualitative and quantitative methods

## Abstract

**Aims:** The modern medical model has been transformed into a biopsychosocial model. The integration of the biopsychosocial approach in healthcare can help improve the effectiveness of diagnosis and treatment. This study explored the actual application of the biopsychosocial approach in healthcare and provides a basis for targeted interventions to promote the biopsychosocial approach in healthcare.

**Methods:** Study 1 involved one-on-one interviews with 30 medical staff and focus group interviews with 16 recent patients. Study 2 was a cross-sectional survey of 13,105 medical staff in Hangzhou, China that analyzed the status quo implementation of the biopsychosocial approach in healthcare.

**Results:** Study 1 found that medical staff did not welcome patients to report information unrelated to their disease, hoping patients did not express their emotions. In the treatment process, patients believed that medical staff refused to attend to or did not encourage reporting of any information other than the disease, and that patients should have reasonable expectations for medical staff. Study 2 found that medical staff had a 37.5% probability of actively paying attention to the patient's psychosocial status. Female medical staff (38.5%) were actively concerned about the patient's psychosocial status significantly more than male medical staff (34.2%) (*P* < 0.01). The medical staff in the psychiatric department (58.4%) paid more active attention to the patient's psychosocial status than staff in the non-psychiatric departments (37.2%). Gender, department, hospital level, and professional title were the factors associated with the medical staff's attention to the patient's psychosocial status (*P* < 0.05). The influence of age on the probability of medical staff actively paying attention to the psychosocial status of patients increased with the number of years of employment. Participants that were 31–40 years old, had an intermediate professional title, and 11–15 years of employment were the least likely to actively pay attention to patients' psychosocial status.

**Conclusion:** Although the biopsychosocial approach has been popularized for many years, it has not been widely used in medical care. Medical staff pay more attention to patients' physical symptoms and less attention to patients' psychosocial status. It is recommended that training will be provided to medical personnel on implementing a biopsychosocial approach with particular attention to the sociodemographic characteristics of medical personnel. Additionally, we propose helping patients set reasonable expectations, and formulating guidelines for implementing the biopsychosocial approach.

## Introduction

With the changes in the spectrum of human diseases, understanding psychological disorders and symptoms continue to deepen. People have become increasingly aware that no single reason could cause the appearance of symptoms, and psychological and social factors need to be considered. Therefore, a purely biomedical model cannot serve and meet the needs of contemporary medical care. In 1977, Engel (1) pointed out the limitations of the biomedical model, integrated psychological and social dimensions, and proposed a biopsychosocial approach. Engel held the view that disease is the result of the interaction of biological, psychological, and social subsystems on multiple levels and highlighted the indispensable role of psychosocial factors, which explained such phenomena as the effect of living conditions on the development of the disease. The premise of the biopsychosocial approach is that the patient's disease cannot be divorced from his or her psychosocial causes, personality, and surroundings (2). Evidence has shown that social/environmental and psychological factors matter in the development of psychiatric disorders (3). In the following decades, the biopsychosocial approach was mentioned in many disciplines and practical fields, including medical traumatic stress, anorexia nervosa, addiction treatment, daily pain, elderly frailty, disability, and health psychology (4–10). With the recognition that some risk factors of the disease are psychosocial rather than biomedical, and that some non-pharmacologic and non-surgical treatment modalities have a therapeutic effect, the biopsychosocial approach potentially improves clinical outcomes for chronic diseases and functional illnesses seen in primary care (11). The biopsychosocial approach in healthcare can improve the effectiveness of diagnosis and treatment (12), which enhances patient satisfaction and can ease conflicts between doctors and patients.

In clinical training such as medical schools and graduate schools, the biopsychosocial approach has been widely taught to arouse attention to the interaction between various factors that affect health and cause diseases (13). However, integrating the biopsychosocial approach into healthcare practice has not been as successful as integrating the approach into research and medical education (14). Most modern healthcare is still based on the biomedical model of disease, which can help identify and treat many diseases. However, it has difficulty recognizing the multi-factor and complexity of many (including non-organic) diseases. In addition, it is easy to ignore the psychosocial status of patients, which may trigger unnecessary disease behaviors in patients (15). Although the biomedical model promotes many healthcare innovations, a biomedical perspective alone cannot guarantee favorable results, nor can it explain the placebo effect and health gaps. It must also include psychological and social factors (10). Suls and Rothman (16) proposed that applying the biopsychosocial framework has not been fully utilized and should be considered in health psychology theories and clinical practice. Fava and Sonino (17) pointed out that although the biopsychosocial framework has been implemented for many years and the evidence base has grown over time, the implementation of this framework in healthcare has been slow. According to Adler (14), studies have found that many medical staff, such as the staff in pain clinics and on medical psychiatric wards, do not adhere to Engel's biopsychosocial approach. The application of the biopsychosocial approach needs thorough evaluation of the psychological, behavioral, sociocultural, and spiritual dimensions of patients' problems, which is time-consuming (18). For physicians who are already overburdened with clinical, administrative, and possibly research tasks, it is a formidable task (11).

However, as diseases become more complicated and multi-factorial, studying the status quo implementation of the biopsychosocial approach has far-reaching significance in health care. Surprisingly, we know very little about the practical application of the biopsychosocial approach in healthcare. To fill this gap in the literature, we explored the practical application of the biopsychosocial approach in healthcare through qualitative and quantitative research methods. Qualitative research in this area can provide us with valuable and comprehensive information and deepen our understanding of the practical application of the biopsychosocial approach in healthcare. Carrying out large-scale quantitative research complements the qualitative data, by investigating and analyzing the current status of implementing the biopsychosocial approach in healthcare and related factors. The knowledge gained would provide a scientific basis for how to carry out effective interventions to promote the status quo implementation of the biopsychosocial approach in healthcare. Specifically, the study could serve as a reference and provide direction for promoting doctor-patient communication; improving patient participation, acceptance, and compliance; improving the effectiveness of diagnosis and treatment; and promoting the harmony in the doctor-patient relationship.

The primary purpose of this study was to explore the implementation of biopsychosocial approach in healthcare and any differences associated with the psychosocial status of patients with different sociodemographic variables. One important factor is the gender of the medical professional. During the consultation process, female doctors have been shown to provide a longer consultation time than male doctors (19). In addition, women were found to use more emotion-focused coping strategies than men (20). Therefore, female doctors may pay more attention to patients' emotions and social factors. Thus, we examined the following hypothesis.

H1: Female medical staff pay more attention to the psychosocial status of patients than male medical staff.

The organizations in which people work affect their thoughts, feelings, and actions in the workplace (21). Hence, the difference in working environments may affect the thoughts and behaviors of medical staff. In a psychiatric department, because the working environment involves patients with mental illness, psychiatric staff may pay more attention to the psychosocial status of patients than in an environment where the medical staff are working with non-psychiatric patients. Therefore, we examined the following hypothesis.

H2: Psychiatric medical staff pay more attention to the psychosocial status of patients than non-psychiatric medical staff.

In China, hospitals are divided into three levels according to their functions and tasks (22). The first-level hospitals provide the community with primary healthcare, prevention, rehabilitation, and health care services. The second-level hospital is responsible for providing diagnosis and treatment of common and frequently occurring diseases for the community. The tertiary hospital is a comprehensive medical institution that provides specialized medical services (23). Medical staff at different hospital levels face different workloads, different kinds or parts of training, and different working environments, which may affect their attention to the psychosocial status of patients. Among them, tertiary hospitals provide diagnosis and treatment services for acute, critical, and difficult and complex diseases, which require comprehensive evaluation of patients. As such, medical staff in tertiary hospitals may pay more attention to the psychosocial status of patients than medical staff in secondary hospitals and first-level hospitals. Consequently, we proposed the following hypothesis.

H3: Medical staff in tertiary hospitals pay more attention to the psychosocial status of patients than medical staff in first- and second-level hospitals.

Lastly, it has been shown that burnout symptoms among doctors are prevalent and associated with age, professional title, and long working hours (24). Age and years of employment are related to the psychosocial workload of medical staff (25), which may affect the attention of medical staff to the psychosocial status of patients. Medical staff may face pressure from job tasks and their promotion to professional titles, and the professional title may affect their attention to the psychosocial status of patients. Medical staff with senior professional titles may pay more attention to the psychosocial status of patients. However, medical staff with junior and intermediate titles are faced with heavy workloads and the pressure to be promoted. Therefore, they may pay less attention to the psychosocial aspects of patients. Given these differences, our last hypothesis was as follows:

H4: Medical staff's attention to patients' psychosocial status will be associated with their age, years of employment, and professional title.

## Materials and Methods

### Design and Participants

This research study used a combination of qualitative and quantitative research methods. Study 1 conducted one-on-one interviews with 30 medical staff and conducted focus group interviews with 16 recent patients to summarize the views of both doctors and patients on the biopsychosocial approach. Participants in the one-on-one interviews were selected through random sampling from medical staff in the outpatient and ward areas of a large tertiary hospital in Zhejiang, China in September 2019. The researcher introduced himself to the interviewees who met the inclusion criteria and explained the purpose and methods of the study. After obtaining consent, the interview was conducted according to a semi-structured interview outline determined in advance. The interview began by asking for basic information on the participant, such as department and years of employment, which was followed by the interview questions, such as “What information do you want the patient to tell you when you are providing treatment?” and “What about the patient's behavior do you think will hinder the diagnosis and treatment?” The participants included 13 men and 17 women. Their average working experience was 9.84 ± 8.08 years and they were from diverse medical fields (e.g., internal medicine, urology, endocrinology).

Two focus group interviews were conducted in June 2020, and each group included eight participants. A semi-structured interview outline was prepared in advance for the purposes of the group interview, which asked the participants to “Please talk about your most recent medical experience,” and questions such as “During the treatment, what behaviors or reactions do you think will promote or hinder the medical treatment process?” Inclusion criteria for the focus group were clear verbal expression and medical experience in the past 6 months. The participants were 6 men and 10 women with an average age of 22.9 ± 2.11 years. The researcher introduced himself to the patients who met the inclusion criteria and explained the purpose and methods of the research. The researcher obtained informed consent from each participant before conducting the focus group. Focus group interviews were recorded and the researcher took notes.

From December 2020 to January 2021, Study 2 was carried out in Hangzhou, Zhejiang Province. An anonymous online questionnaire was used to gather data on the current status of implementing the biopsychosocial approach in healthcare. The questionnaire asked for demographic information including gender, department, hospital level, professional title, age, years of employment, and the probability of actively paying attention to the patients' psychosocial status. To assess the probability of medical staff actively paying attention to the patient's psychosocial status, participants were asked, “During the consultation process, in ()% of the cases, I will actively pay attention to the patient's psychosocial status rather than just the physical symptoms.” A total of 13,105 medical staff were surveyed.

Table 1 shows detailed information on the participants' characteristics. Of the 13,105 eligible medical staff that were included in this study, 23.5% (*n* = 3,084) were men and 76.5% (*n* = 10,021) were women. A total of 1.6% (*n* = 206) were psychiatric medical staff and 98.4% (*n* =12,899) were non-psychiatric. There were 2,681 (20.5%) medical staff from tertiary hospitals, 5,064 (38.6%) medical staff from second-level hospitals, and 5,360 (40.9%) medical staff from first-level hospitals. Approximately half (50.8%) of the medical staff had junior titles and 4,627 (35.3%) had intermediate titles. In terms of age, 27.6% (*n* = 3,614) were ages 20–30, 41.7% (*n* = 5,466) were 31–40, 20.3% (*n* = 3,018) were 41–50, and 7.7% (*n* = 1,007) were ages 51 or older. With regard to years of employment, 19.6% (*n* = 2,567) were employed 0–5 years, 19.9% (*n* = 2,603) 6–10 years, 25.2% (*n* = 3,305) 11–15 years, 9.8% (*n* = 1,289) 16–20 years, and 25.5% (*n* = 3,341) were employed for 21 years or more.

**Table 1:** The descriptive characteristics of the participants.

**Demographic characteristics**	***n* (%)**	***M***	**SD**	***t***	***P***
**Gender**					
Male	3,084 (23.5)	34.2	30.4	−6.8	0.00
Female	10,021 (76.5)	38.5	31.0		
**Department**					
Psychiatric	206 (1.6)	58.4	33.1	−9.8	0.00
Non-psychiatric	12,899 (98.4)	37.2	30.8		
**Hospital level**					
Tertiary hospital	2,681 (20.5)	42.6	32.6	94.5	0.00
Second-level hospital	5,064 (38.6)	39.2	31.3		
First-level hospital	5,360 (40.9)	33.3	29.2		
**Professional title**					
Junior	6,662 (50.8)	40.1	31.9	35.5	0.00
Intermediate	4,627 (35.3)	34.1	29.5		
Deputy Senior	1,412 (10.8)	36.5	30.1		
Senior	404 (3.1)	37.8	30.6		
**Age**					
20–30 years	3,614 (27.6)	42.4	32.1	53.3	0.00
31–40 years	5,466 (41.7)	34.3	30.0		
41–50 years	3,018 (23.0)	36.4	30.4		
>50 years	1,007 (7.7)	40.6	31.0		
**Years of employment**					
0–5 years	2,567 (19.6)	43.1	32.1	34.9	0.00
6–10 years	2,603 (19.9)	37.6	31.3		
11–15 years	3,305 (25.2)	33.8	30.0		
16–20 years	1,289 (9.8)	35.4	30.0		
>20 years	3,341 (25.5)	37.6	30.4		

*The M and SD in this table are the mean and its associated standard deviation of the probability of medical staff actively paying attention to the patient's psycho-social state*.

### Statistical Analyses

In Study 1, we used thematic analysis to analyze the qualitative data. Initially, we transcribed the recorded interview then reviewed the transcribed data three times to obtain a general understanding. Next, we extracted semantic units and classified them as compact units. We then honed the important parts of each unit and what aspects of the qualitative data it covered. Next, the compact unit was further summarized and marked with appropriate headings. In addition, we searched for overlapping areas between topics, identified emerging subtopics, provided more detailed topic descriptions and described the hierarchical structure in the data, and clearly defined the scope of each topic. Finally, the sub-categories were grouped according to similarities and differences, and appropriate titles that could represent the resulting categories were selected.

In Study 2, we analyzed the sociodemographic variables and calculated the number and percentage distribution of the categorical variables. The independent *t*-test and one-way analysis of variance were used to determine sociodemographic differences among medical staff with regard to actively paying attention to patients' psychosocial status. Lastly, all variables were included in a stepwise linear regression model (the entry/clearance criterion was *P* = 0.05/0.1) for analysis. All statistical analyses were performed using IBM SPSS Version 26.0, and *P* < 0.05 (two-tailed) was considered statistically significant.

## Results

### Study 1

#### Medical Staff Do Not Welcome Patients to Report Information That Is Not Related to the Disease

During the consultation process, some medical staff paid more attention to the patient's physical symptoms. Patients were not welcome to report information that was not related to the disease. Medical staff hoped that the patient would grasp the key points when explaining their condition.

“The patients only need to talk about the disease and what is related to the disease during the communication with medical staff, and not mention other content.” (A male orthopedic doctor who has worked for 10 years)

“During the treatment, the patient does not need to say too much that has nothing to do with the symptom.” (A female doctor in the urology department who has worked for 7 years)

“I hope that the patient's parents can accurately provide the child's medical history and clearly describe the condition.”(A female neonatologist who has worked for 7 years)

“The patient should focus on the critical points in the process of describing the condition.” (A female internal medicine outpatient doctor who has worked for 1 year)

#### Medical Staff Hope That Patients Will Not Confide in Them

The medical staff said that although they can understand the patients' mood, they hoped that the patient would not confide their emotions to them and that they need to a maintain a rational attitude.

“Although the patient's mood is understandable, the patient does not need to say many things that have nothing to do with the patient's condition and only need to answer my questions accurately.” (A respiratory physician who has worked for 9 years)

“I hope that the patients will not confide their emotions to the medical staff.” (A female doctor in the gastroenterology department who has worked for 3 years)

“The patient's anxiety is understandable, but the patient should maintain a rational attitude during the treatment process.” (A female doctor in the endocrinology department who has worked for 8 years)

#### The Patient Felt That Medical Staff Refused to Pay Attention to the Patient's Psychosocial Status

Some patients expressed their desire to get the attention of medical staff, thinking that the medical staff refused to pay attention to the patient's psychosocial status, which made patients feel dissatisfied.

“I want to describe my symptoms perfectly to the medical staff, but the medical staff seems to know me well, and the medical staff do not let me say too much. I feel a little dissatisfied. I want to talk to the medical staff, but the medical staff refuse to understand me.” (Patient Y, male)

“The patients are eager to get the kind of attention from the medical staff. But if there is no particular situation, the medical staff will not pay attention to the patient deliberately.” (Patient Z, female)

“I feel obstructed when communicating with some doctors, and the doctors may not listen carefully to what I say.” (Patient C, female)

#### Patients Should Have Reasonable Expectations for Medical Staff

Most doctors believed that patients' high expectations would impact the effectiveness of diagnosis and treatment, so patients should have reasonable expectations. Some patients held the view that the patient's expectations for medical staff should be reasonable.

“Excessive expectations of patients have an impact on the effectiveness of diagnosis and treatment. I hope patients have reasonable expectations.” (A male dentist who has worked for 7 years)

“Medical staff have as part of their responsibilities to take care of patients' emotions, but do not expect clinical medical staff to comfort patients like psychological medical staff.” (Patient A, female)

“Patients are emotionally sensitive, which may hinder the doctor's diagnosis and treatment. Sometimes patients need to control their emotions and calm their minds to some extent.” (Patient D, female)

### Study 2

#### Comparison of the Probability of Medical Staff Actively Paying Attention to the Psychosocial Status of Patients

There were significant differences in the probability of actively paying attention to the psychosocial status of patients according to gender, department, hospital level, professional title, age, and years of employment (*P* < 0.01). Female medical staff (38.5%) were more likely to pay attention to the psychosocial status of patients than male medical staff (34.2%) (*P* < 0.01). The medical staff in the psychiatry department (58.4%) paid more attention to patients' psychosocial status than the medical staff in other departments (37.2%).

Table 2 provides the results of the comparisons according to hospital level, professional title, age, and years of employment. The probability of medical staff in second-level and tertiary hospitals actively paying attention to the psychosocial status of patients was significantly higher than that of medical staff in first-level hospitals, and medical staff in tertiary hospitals were more likely to pay attention to psychosocial status than medical staff in second-level hospitals.

**Table 2:** Comparison of medical staff in different demographic characteristics actively paying attention to the psycho-social state of patients.

**Demographic characteristics**		**Mean difference (I-J)**	**SE**	***P***	**CI**
**Hospital level**					
Tertiary hospital	Second-level hospital	3.5	0.8	0.00	(1.66, 5.26)
	First-level hospital	9.3	0.8	0.00	(7.56, 11.06)
Second-level hospital	Tertiary hospital	−3.5	0.8	0.00	(−5.26, −1.66)
	First-level hospital	5.9	0.6	0.00	(4.46, 7.25)
First-level hospital	Tertiary hospital	−9.3	0.8	0.00	(−11.06, −7.56)
	Second-level hospital	−5.9	0.6	0.00	(−7.25, −4.46)
**Professional title**					
Junior	Intermediate	6.0	0.6	0.00	(4.49, 7.49)
	Deputy Senior	3.6	0.9	0.00	(1.31, 5.90)
	Senior	2.3	1.6	0.45	(−1.73, 6.37)
Intermediate	Junior	−6.0	0.6	0.00	(−7.49, −4.49)
	Deputy Senior	−2.4	0.9	0.04	(−4.73, −0.05)
	Senior	−3.7	1.6	0.09	(−7.75, 0.41)
Deputy Senior	Junior	−3.6	0.9	0.00	(−5.90, −1.31)
	Intermediate	2.4	0.9	0.04	(0.05, 4.73)
	Senior	−1.3	1.7	0.88	(−5.71, 3.15)
Senior	Junior	−2.3	1.6	0.45	(−6.37, 1.73)
	Intermediate	3.7	1.6	0.09	(−0.41, 7.75)
	Deputy Senior	1.3	1.7	0.88	(−3.15, 5.71)
**Age**					
20–30	31–40	8.1	0.7	0.00	(6.37, 9.81)
	41–50	6.1	0.8	0.00	(4.07, 8.02)
	>50	1.8	1.1	0.36	(−1.05, 4.68)
31–40	20–30	−8.1	0.7	0.00	(−9.81, −6.37)
	41–50	−2.0	0.7	0.02	(−3.81, −0.28)
	>50	−6.3	1.1	0.00	(−9.00, −3.55)
41–50	20–30	−6.1	0.8	0.00	(−8.02, −4.07)
	31–40	2.0	0.7	0.02	(0.28, 3.81)
	>50	−4.2	1.1	0.00	(−7.12, −1.34)
>50	20–30	−1.8	1.1	0.36	(−4.68, 1.05)
	31–40	6.3	1.1	0.00	(3.55, 9.00)
	41–50	4.2	1.1	0.00	(1.34, 7.12)
**Years of employment**					
0–5	6–10	5.54	0.9	0.00	(3.14, 7.95)
	11–15	9.3	0.8	0.00	(7.10, 11.58)
	16–20	7.7	1.1	0.00	(4.83, 10.57)
	>20	5.6	0.8	0.00	(3.33, 7.83)
6–10	0–5	−5.5	0.9	0.00	(−7.95, −3.14)
	11–15	3.8	0.8	0.00	(1.59, 5.99)
	16–20	2.2	1.0	0.23	(−0.68, 4.99)
	>20	0.0	0.8	1.00	(−2.17, 2.24)
11–15	0–5	−9.3	0.8	0.00	(−11.58, −7.10)
	6–10	−3.8	0.8	0.00	(−5.99, −1.59)
	16–20	−1.6	1.0	0.46	(−4.33, 1.06)
	>20	−3.8	0.7	0.00	(−5.78, −1.73)
16–20	0–5	−7.7	1.1	0.00	(−10.57, −4.83)
	6–10	−2.2	1.0	0.23	(−4.99, 0.68)
	11–15	1.6	1.0	0.46	(−1.06, 4.33)
	>20	−2.1	1.0	0.20	(−4.82, 0.58)
>20	0–5	−5.6	0.8	0.00	(−7.83, −3.33)
	6–10	−0.0	0.8	1.00	(−2.24, 2.17)
	11–15	3.8	0.7	0.00	(1.73, 5.78)
	16–20	2.1	1.0	0.20	(−0.58, 4.82)

*The CI means a 95% probability that the confidence interval contains the overall mean. The probability of correct estimation is 0.95, and the probability of estimation error is 0.05*.

The probability of medical staff 20–30 years old actively paying attention to the patient's psychosocial status was significantly higher than that of medical staff 31–40 years old and those who were 41–50 years old. Medical staff aged 41–50 and over 50 were more likely to pay attention to the psychosocial status of patients than those who were 31–40. The medical staff over the age of 50 were more likely to actively pay attention to the psychosocial status of patients than those aged 41–50. Medical staff aged 31–40 were the least likely to pay attention to patients' psychosocial status.

The probability of medical staff with junior professional titles actively paying attention to the psychosocial status of patients was significantly higher than that of the medical staff with intermediate professional titles and deputy senior professional titles. The probability that medical staff with deputy senior professional titles and senior professional titles actively pay attention to the psychosocial status of patients was significantly higher than that of the medical staff with intermediate professional titles. Medical staff with intermediate professional titles were the least likely to pay attention to the psychosocial status of patients.

Medical staff who had worked for 0–5 years were more likely to actively pay attention to the psychosocial status of patients than other medical staff who had worked for more years. Medical staff who had worked for 6–10 years were more likely to pay attention to the psychosocial status of patients than those who had worked for 11–15 years. Medical staff who had worked for more than 21 years were more likely to pay attention to the psychosocial status of patients than those who had worked for 11–15 years. Medical staff who had worked for 11–15 years were the least likely to actively pay attention to the psychosocial status of patients.

#### Regression Analysis on the Probability of Medical Staff Actively Paying Attention to the Psychosocial Status of Patients

In order to identify the statistical significant characteristics of medical staff who actively pay attention to the psychosocial status of patients, we first included all sociodemographic variables into the stepwise linear regression analysis. Gender was indexed as 0 = male, 1 = female. Then, considering the possible interaction between hospital level and professional title, and between age and years of employment, we included the interaction terms “hospital level × professional title” and “age × years of employment” into the regression equation. The results are shown in Table 3. Gender [β = 0.05, CI (2.66, 5.19), *P* < 0.01], department [β = 0.07, CI (13.48, 21.93), *P* < 0.01], hospital level [β = 0.10, CI (3.51, 4.92), *P* < 0.01], and professional title [β = −0.04, CI (−2.41, −0.68), *P* < 0.01] were statistical significant predictors of the probability of medical staff actively paying attention to the psychosocial status of patients. Age and years of employment were not statistical significant. Age and working years cannot independently predict the probability of medical staff actively paying attention to the psychosocial state of patients. But the interaction of age and years of employment was statistical significant [β = 0.11, CI (0.03, 0.04), *P* < 0.01]. The influence of age on the probability of medical staff actively paying attention to the psychosocial status of patients increased with the increase in years of employment. The interaction of hospital level and professional title level was not statistical significant.

**Table 3:** Linear regression analysis of the probability of medical staff actively paying attention to the psycho-social state of patients.

**Variable**	**β**	**SE**	***t***	***P***	**CI**
Gender	0.054	0.645	6.081	0.000	(2.660, 5.190)
Department	0.071	2.155	8.214	0.000	(13.477, 21.926)
Hospital level	0.103	0.361	11.669	0.000	(3.507, 4.923)
Professional title	−0.039	0.443	−3.484	0.000	(−2.412, −0.675)
Age	−0.003	0.076	−0.154	0.877	(−0.161, 0.137)
Years of employment	−0.043	0.072	−1.941	0.052	(−0.282, 0.001)
Hospital level × Professional Title level	−0.017	0.448	−1.950	0.051	(−1.753, 0.005)
Age × Years of employment	0.106	0.003	10.391	0.000	(0.025, 0.036)

*The β is the estimate resulting from an analysis performed on standardized variables, representing the effect of an independent variable on the dependent variable. The SE indicates the deviation between the actual value and the regression estimate due to sampling error. The t is the significance test value of the t-test of the regression coefficient. The CI means a 95% probability that the confidence interval contains the overall mean. The probability of correct estimation is 0.95, and the probability of estimation error is 0.05*.

## Discussion

In this study, we explored the implementation of the biopsychosocial approach in healthcare through a combination of qualitative and quantitative research methods. Our focus was to understand the experience of medical staff and patients with regard to the attention given to the psychosocial status of patients, and determine what sociodemographic factors were associated with differences among medical staff in the active attention they give to patients' psychosocial status. One qualitative research finding was that medical staff do not welcome patients to report information unrelated to the disease and hope that patients will not confide in them. Quantitative research found that medical staff had a 37.5% probability of actively paying attention to the patient's psychosocial status. This shows that medical staff focus on the patient's physical symptoms and tend to ignore the patient's psychosocial status. Based on the sample in the present study, it can be concluded that the biopsychosocial approach is not sufficiently applied in healthcare.

Another finding from the qualitative study was that some patients held the view that medical staff refused to pay attention to the patients' psychosocial status. The patients were eager to get such attention from the medical staff, which is consistent with previous research results. Vinson found that patients increasingly wanted to interact emotionally with medical staff (26). In the eyes of patients, the medical staff take care of the patient's emotions to a certain extent, which helps patients to relax. Patients feel helpless and hopeless in the face of the disease, and medical staff play an essential role in providing support to patients (27). The integration of biopsychosocial methods in healthcare needs to be established within medical staff (14).

In addition, according to the results of the qualitative study, patients should have reasonable expectations of the medical staff. Patients who go to the hospital generally have expectations regarding the care that they will receive. These expectations range from a desire for information or psychosocial support to expectations for specific tests or treatments. Fulfillment of patients' expectations may influence health care utilization, affect patient satisfaction, and be used to indicate quality of care (28). Health care expectations may be positive or negative (29). Particular emphasis should be placed on patients with excessive expectations, as the lack of an achievable balance between expectations and fulfillment may lead to dissatisfaction (30). Therefore, in routine medical services, medical staff should discuss the treatment plan with patients and the realization of short- and long-term goals to ensure that patients' expectations are realistic and reasonable (31). Medical staff should actively listen to determine patients' understanding and concerns about the disease, respond to patients' concerns, and help set reasonable expectations, which is helpful to establish a harmonious doctor-patient relationship.

The quantitative study results verified our first hypothesis. Female medical staff (38.5%) were more likely to actively pay attention to the psychosocial status of patients than male medical staff (34.2%). Our findings are consistent with prior studies indicating that the gender of the doctor is a relevant factor in the differences in medical care provided. For example, it was found that female doctors take an average of 2 min longer to see a patient than male doctors (32), are more likely to ask patients about health risks and unhealthy behaviors and provide more psychological support (33).

In addition, department and hospital level were factors associated with medical staff actively paying attention to the psychosocial status of patients. Psychiatric medical staff actively paid more attention to the psychosocial status of patients than non-psychiatric medical staff. This verifies our hypothesis that psychiatrists would pay more attention to the psychosocial status of patients due to the particularity of the department. However, our results showed that only 58.4% of the psychiatric medical staff paid attention to the patient's psychosocial status. Regarding hospital level, medical staff in tertiary and second-level hospitals were more likely to actively pay attention to the psychosocial status than medical staff in first-level hospitals. To a certain extent, our results are consistent with Meretoja et al.'s (34) finding that competence profiles differed in both the level and infrequency of using competencies according to work environment. There are differences in the work environments of hospitals of different levels, including the competence of the medical staff, which may affect the degree to which medical staff pay attention to the psychosocial status of patients.

The hypothesis that medical staff's active attention to patients' psychosocial status is related to age, years of employment, and professional title was also supported. Professional title and the interaction of age and years of employment had predictive effects on the probability of medical staff actively paying attention to the patient's psychosocial status. The influence of age increased with the increase in years of employment. We found that the medical staff aged 31–40 years, with an intermediate professional title, and 11–15 years of employment were least likely to actively pay attention to the patient's psychosocial status. Previous studies have found that age, working years, and work burden were essential predictors of job burnout for doctors and nurses (35). Compared with other occupations, occupational stress and burnout symptoms were more common among doctors (36). The job burnout of doctors was related to changes in the professional environment, such as financial pressure, increased workload, and index assessment (37). Therefore, we speculate that medical staff aged 31–40 with intermediate professional titles and 11–15 years of employment may have a heavier workload, more tremendous pressure for promotion, and face more severe job burnout. Thus, they have the lowest probability of actively focusing on the patient's psychosocial status.

Based on the findings mentioned above in this study, the biopsychosocial approach has not been widely used in healthcare. Most medical staff tend to only focus on the patients' physical symptoms and not pay attention to the patients' psychosocial status. Hence, it is recommended that the biopsychosocial approach be promoted in medical treatment through training and interventions for medical staff, primarily geared to those with the lowest probability of actively focusing on the patients' psychosocial status. Further, it is suggested to comprehensively popularize the knowledge of medical psychology among medical staff and carry out the research of disease psychology, which will help strengthen the medical staff's attention to the biopsychosocial medical model. Then, in medical practice, guidelines for implementing the biopsychosocial medical model should be formulated so that the patients' disease's biological, psychological, and social components are considered and managed as a whole. For example, medical and psychology departments could establish an efficient consultation, referral, or a multi-disciplinary treatment team to enhance patient diagnosis and treatment.

### Strengths and Limitations

This research explored the integration of the biopsychosocial approach into health care from the perspective of medical staff and patients. Using qualitative and quantitative methods, the study provides comprehensive information and fills a gap in the research on the application of the biopsychosocial approach. The study included a diverse and extensive sample of medical staff, as well as interviewing doctors and patients for their perspective on the issue. Furthermore, the study investigated sociodemographic variables in relation to medical staff's attention to patients' psychosocial status. As such, the study provides scientific evidence for carrying out effective interventions to promote the implementation of the biopsychosocial approach in healthcare.

This study has some limitations. Firstly, although the sample included 13,105 medical staff in Hangzhou City, the results may not be generalizable to the situation of medical staff in other countries and regions. Future research should focus on other countries and regions with comparative analyses. Secondly, Study 2 used self-report questionnaires, which are subject to response bias such as social desirability. Lastly, this is a cross-sectional study which does not show how these variables behave over time. Future studies should consider using longitudinal designs.

## Conclusion

Although the biopsychosocial approach has been popularized for many years, it has not been widely used in medical care. The results of the present study suggest that medical staff tend to focus their attention on the patients' physical symptoms and are less inclined to attend to patients' psychosocial status. Gender, department, hospital level, professional title and the interaction of age and years of employment can play a predictive role in the extent to which medical staff pay attention to patients' psychosocial status. Therefore, it is recommended that training and interventions be provided for medical staff on integrating the biopsychosocial approach into the provision of health care. In developing and implementing any in-service training for medical staff, it would be important to consider how the factors identified in this study may impact the ability and motivation of medical staff to attend to the psychosocial status of patients. Additionally, we propose guidelines be formulated for implementing the biopsychosocial approach, and helping patients set reasonable expectations regarding what the medical staff is able to do given their job responsibilities and the timeframe they have to provide diagnosis and treatment.

## Data Availability Statement

The data that support the findings of this study are available from the corresponding author upon reasonable request.

## Ethics Statement

The studies involving human participants were reviewed and approved by ethical review institutions of Zhejiang University and related hospitals. The participants provided their informed consent to participate in this study.

## Author Contributions

QY, XX, and HS contributed to the conception of the study. HS, TS, and ZW collected the data. YX and XX carried out data cleaning. XX performed the data analyses and wrote the manuscript. QY and HS contributed to critically revising the manuscript for important content. All authors have read and agreed to the published version of the manuscript and contributed to the article and approved the submitted version.

## Funding

This research was funded by National Natural Science Foundation of China (Grant Numbers 71974170), Leading Innovative and Entrepreneur Team Introduction Program of Zhejiang (Grant Numbers 2019R01007) and Public Projects of Science and Technology Department of Zhejiang Province (Grant Numbers LGF21H090006) to HS.

## Conflict of Interest

The authors declare that the research was conducted in the absence of any commercial or financial relationships that could be construed as a potential conflict of interest.

## Publisher's Note

All claims expressed in this article are solely those of the authors and do not necessarily represent those of their affiliated organizations, or those of the publisher, the editors and the reviewers. Any product that may be evaluated in this article, or claim that may be made by its manufacturer, is not guaranteed or endorsed by the publisher.
